# The impact of preschool child and maternal attention-deficit/hyperactivity disorder (ADHD) symptoms on mothers’ perceived chronic stress and hair cortisol

**DOI:** 10.1007/s00702-021-02377-1

**Published:** 2021-07-06

**Authors:** Anna Szép, Nadine Skoluda, Susan Schloß, Katja Becker, Ursula Pauli-Pott, Urs M. Nater

**Affiliations:** 1 Clinical Psychology of Childhood and Adolescence, Department of Psychology, Philipps-University of Marburg, Gutenbergstraße 18, 35032 Marburg, Germany; 2 Department of Clinical and Health Psychology, Faculty of Psychology, University of Vienna, Liebiggasse 5, 1010 Vienna, Austria; 3 Department of Child and Adolescent Psychiatry, Psychosomatics and Psychotherapy, Philipps-University of Marburg, Hans Sachs Str. 6, 35039 Marburg, Germany; 4 Center for Mind, Brain and Behavior (CMBB), University of Marburg and Justus Liebig University Giessen, Giessen, Germany

**Keywords:** Hair cortisol, Chronic stress, ADHD

## Abstract

Providing care for a child with attention-deficit/hyperactivity disorder (ADHD) is associated with parenting stress. Moreover, adults with elevated ADHD symptoms report increased perceived stress. Despite this, it has rarely been examined whether and how child and maternal ADHD symptoms may affect maternal perceived stress and the stress-sensitive hypothalamic–pituitary–adrenal axis. This study therefore investigated the possible impact of child and maternal ADHD symptoms on mothers' perceived chronic stress and hair cortisol concentration (HCC), while simultaneously considering the effects of child oppositional defiant/conduct disorder (ODD/CD) and maternal depressive symptomatology. In total, 124 mothers (35.96 ± 5.21 years) of preschool children were included. Maternal perceived stress, ADHD and depressive symptoms were assessed using self-report measures. Child ADHD symptoms were assessed using an interview and questionnaires completed by mothers and teachers. Additionally, mothers provided information about their children’s ODD/CD symptoms. Hair samples were taken from mothers to assess HCC. Child and maternal ADHD, child ODD/CD, and maternal depressive symptoms accounted for 50% of the variance in perceived chronic stress (*F*(4, 119) = 30.24; *p* < 0.01), with only maternal ADHD (*β* = 0.52, *p* < 0.01) and depressive symptoms (*β* = 0.49, *p* < 0.01) being uniquely significant. Maternal ADHD symptoms did not moderate the relationship between child ADHD symptoms and maternal perceived chronic stress (*b* = − 0.01; SE* b* = 0.17; *t*(5, 118) = − 0.05; *p* = 0.96). Mother’s age became the only significant predictor of maternal HCC (*β* = 0.29; *p* < 0.01). Based on these findings, practitioners are advised to be aware of and take into account possible maternal ADHD and depressive symptomatology and perceived chronic stress when treating children diagnosed with ADHD.

**Supplementary Information:**

The online version contains supplementary material available at 10.1007/s00702-021-02377-1.

## Introduction

Providing care for a significant other with special needs, such as caring for a relative with dementia (Gouin et al. 2012), intellectual disability (Peer and Hillman 2014), or autism spectrum disorder (ASD) (Radin et al. 2019), has been associated with elevated perceived chronic stress. As such, being an informal caregiver^1^ of a family member with a chronic disorder is associated with significant chronic stress and potentially severe negative health implications (Roth et al. 2015). While previous studies have provided evidence that parents of children with attention-deficit/hyperactivity disorder (ADHD) experience a substantial amount of parenting stress (Johnston and Mash 2001; Theule et al. 2013), self-reported chronic stress in this caregiver population has rarely been investigated.

ADHD is one of the most common psychiatric disorders, affecting approximately 5% of school-aged children (APA 2013; Feldman and Reiff 2014; Polanczyk et al. 2007). It comprises symptoms of inattention, hyperactivity, and impulsivity. These three core symptoms are age-inappropriate, pervasive (occurring in more than one important life domain), and lead to severe functional impairments in different settings, e.g. school or family (APA 2013; Feldman and Reiff 2014). Caregivers of children with ADHD face demands that exceed those of parents of children without a chronic disorder (Johnston and Mash 2001). Due to their children’s constant care needs, caregivers experience considerable time constraints, social isolation as well as difficulties in their employment (Kvist et al. 2013; Sikirica et al. 2015). Although ADHD is usually diagnosed in school age (Wolraich et al. 2019), its symptoms and associated impairments (poor social and academic functioning) appear as early as preschool age (Egger et al. 2006), placing strain on parents of children with ADHD early on.

ADHD is often accompanied by comorbid disorders (Wolraich et al. 2019). Oppositional defiant and conduct disorder (ODD/CD) are among the most common comorbid conditions, affecting about half of paediatric ADHD patients (Connor et al. 2010). Moreover, ODD/CD symptoms in children with ADHD have been shown to increase their parents’ parenting stress (Anastopoulos et al. 1992; Theule et al. 2013; Wiener et al. 2016). While some studies suggest that both child ADHD and ODD/CD symptoms contribute to parenting stress (Anastopoulos et al. 1992; Munoz-Silva et al. 2017), others reported diminished effects of child ADHD symptoms on parenting stress of mothers after including ODD/CD symptoms (Schworer et al. 2020; Theule et al. 2011). Thus, in order to disentangle whether mothers’ chronic stress is affected by child ADHD or rather by comorbid ODD/CD symptoms, it is necessary to control for comorbid ODD/CD symptoms.

Although current classification systems characterise ADHD as a categorical disorder, more and more studies indicate that ADHD symptoms constitute a spectrum on which ADHD symptoms are dimensionally distributed. Individuals on the higher end of the spectrum, even if they do not meet all the diagnostic criteria of ADHD, display pervasive functional impairments in everyday life (Balázs and Keresztény 2014; Heidbreder 2015). Hence, the examination of a broad spectrum of individuals with high levels of ADHD symptoms would enable a better understanding of the negative impacts that ADHD symptoms exert on an individuals life.

ADHD is a heritable disorder, with 18–44% of children with ADHD having a parent who has experienced ADHD during childhood and/or adulthood (Biederman 2005; Sprich et al. 2000; Starck et al. 2016). Previous studies have revealed substantial evidence for an association between adult ADHD symptoms and perceived general stress (Combs et al. 2015; Hirvikoski et al. 2009). Moreover, parental ADHD symptomatology has also been shown to be related to parenting stress (Biondic et al. 2019; Schworer et al. 2020; Theule et al. 2011; Wiener et al. 2016). Indeed, in some of these studies, the effect of child ADHD symptoms became non-significant after controlling for maternal ADHD symptoms (Theule et al. 2011; Wiener et al. 2016). Breaux and Harvey (2019), on the other hand, found a significant effect of child ADHD symptoms on mothers’ general life stress even after controlling for child ODD/CD and maternal ADHD symptoms. Therefore caregivers’ ADHD symptoms should be considered as an important factor contributing to mothers’ perceived chronic stress.

ADHD in adulthood is often accompanied by comorbid depression (Fayyad et al. 2017). Depressive symptoms are more prevalent in mothers of children with ADHD compared to control families (Chronis et al. 2003; Johnston and Mash 2001) and have consistently been shown to have a large effect on parenting stress in mothers of children with ADHD (Anastopoulos et al. 1992; Biondic et al. 2019; Schworer et al. 2020; Theule et al. 2013). Studies examining the simultaneous effects of maternal ADHD and depressive symptoms on parenting stress found that both of these factors contributed to mothers’ parenting stress (Biondic et al. 2019; Schworer et al. 2020). The first aim of the present study, is therefore, to investigate the relative contribution of child and maternal ADHD symptoms on maternal perceived chronic stress, while simultaneously considering the effects of child ODD/CD and maternal depressive symptoms.

It is possible that maternal ADHD symptoms do not merely add to the effect of child ADHD symptoms, but rather moderate the impact thereof. Study findings from the field of parenting research suggest that maternal ADHD symptoms either mitigate or exacerbate the negative impact of child ADHD symptoms on parenting behaviour (Psychogiou et al. 2008). Although the model of Psychogiou et al. (2008) was originally developed with respect to parenting behaviour, it is conceivable that this model is also applicable to maternal perceived chronic stress. In terms of the way in which maternal ADHD symptoms may moderate the negative effects of child ADHD on maternal perceived chronic stress, two competing hypotheses can be derived and transferred to the field of stress research from the work of Psychogiou et al. (2008).

The modified *similarity-misfit hypothesis* proposes that child ADHD symptoms might have a greater impact on perceived chronic stress in mothers with elevated ADHD symptoms compared to those with low ADHD symptoms. This assumption is based on previous findings showing higher levels of conflict in families in which both mothers and children exhibit elevated ADHD symptoms (Agha et al. 2013; Grimbos and Wiener 2018). It is assumed that these increased conflict situations are stressful and are likely to further increase perceived stress in mothers with elevated ADHD symptoms.

The alternative and modified *similarity-fit hypothesis* predicts that the adverse effects of child ADHD symptoms on maternal perceived chronic stress might be ameliorated by maternal ADHD symptoms. Psychogiou et al. (2008) suggested that mothers with high levels of ADHD symptoms are likely to be tolerant and empathetic towards their children's ADHD-induced behaviour due to their own ADHD history, and interact smoothly with their children due to their shared behavioural style (Psychogiou et al. 2008). In turn, this may have a dampening impact on mothers’ perceived chronic stress. The similarity-fit hypothesis is supported by a study reporting reduced irritability in mothers with elevated ADHD symptoms toward their children with ADHD (Griggs and Mikami 2011). In another study, maternal ADHD symptoms were positively associated with parenting stress in mothers of children without ADHD but not in mothers of children with ADHD (Perez Algorta et al. 2018), indicating that child ADHD symptoms may not affect parenting stress in mothers with elevated ADHD symptoms.

Thus, the second aim of this study is to test the similarity-fit/misfit hypothesis in the context of maternal perceived chronic stress.

Chronic stress not only induces psychological changes but also affects the physiology of the body (McEwen 2005). The hypothalamic–pituitary–adrenocortical (HPA) axis is one of the major endocrine systems involved in the physiological stress reaction (McEwen 1998). Accordingly, the determination of HPA axis activity has emerged as an important biological correlate and measure of stress (Miller et al. 2007).

The chronic stress of caregiving has been associated with altered HPA axis activity. More specifically, a hypoactive HPA axis has been found in the caregivers of adult offspring with ASD (Seltzer et al. 2010), eating disorders (Ruiz-Robledillo et al. 2016), and schizophrenia (Gonzalez-Bono et al. 2011). However, these studies used salivary cortisol samples to determine HPA axis activity. Salivary cortisol provides a snapshot measure of momentary HPA axis activity and is thus suitable to study short-term changes in cortisol secretion (e.g. stress reactivity, circadian rhythmicity). It is limited, though, when it comes to assessing HPA axis activity over prolonged periods of time, as it requires multiple sampling (resulting in increased material costs and participant burden) and is susceptible to situational factors (such as circadian rhythm or external factors) (Russell et al. 2012). Given the notion that exogenous and endogenous substances cumulatively incorporate into the hair shaft, hair cortisol concentration (HCC) constitutes an alternative measure to assess long-term cortisol exposure reliably and retrospectively (Stalder et al. 2017).

To date, only one study has used HCC to examine HPA axis activity in caregivers of children with a chronic disorder, suggesting a hypoactive HPA axis in mothers of children with ASD (Radin et al. 2019). However, these findings cannot be generalised to caregivers of children with ADHD.

Evidence for a dysregulation of the HPA axis has also been found in individuals with ADHD. Specifically, a meta-analysis of 22 studies examining children reported a significant but weak association between low basal salivary cortisol secretion and ADHD (Scassellati et al. 2012). Longitudinal measurement of HCC revealed that low HCC in preschool age predicted an increase in ADHD symptoms between preschool and school age; this negative association between ADHD and HCC was driven by boys (Pauli-Pott et al. 2017, 2019). However, other studies using salivary cortisol and/ or HCC measures failed to show an association between ADHD and HPA axis activity (Buske-Kirschbaum et al. 2019; Pesonen et al. 2011).

The association between ADHD and HPA axis activity has been scarcely investigated in adult subjects. A meta-analysis of three studies comparing adults with ADHD and controls suggested no difference in salivary cortisol (Bonvicini et al. 2016). However, given the small number of studies, it is only possible to draw preliminary conclusions about HPA axis activity in adults with ADHD. It is conceivable that the stressful nature of experiencing ADHD in combination with perceived stress due to providing care for a child with ADHD might also be associated with alterations of HPA axis activity. Thus, both child and caregiver ADHD symptoms should be considered when analysing integrated cortisol secretion in caregivers of children with ADHD. Given that both child ODD/CD and maternal depressive symptoms might affect maternal perceived chronic stress (Theule et al. 2013), these potentially confounding variables should also be considered in terms of HCC. In sum, the final aim of the current study is to fill a research gap by investigating HCC in primary caregivers of children with ADHD, while considering the effects of child ODD/CD symptoms and maternal ADHD and depressive symptoms.

This study therefore set out to test the following hypotheses: (1) Child and maternal ADHD symptoms predict maternal perceived chronic stress when controlling for child ODD/CD and maternal depressive symptoms; (2) The relationship between child ADHD symptoms and maternal perceived chronic stress is moderated by maternal ADHD symptoms; (3) Child and maternal ADHD symptoms predict maternal HCC when controlling for child ODD/CD and maternal depressive symptoms.

## Methods

### Participants

The present study draws on data from the first assessment wave of an ongoing longitudinal study (Pauli-Pott et al. 2017, 2019; Schloss et al. 2019) following the development of 4–5-year-old children with and without ADHD symptoms. A community-based sample was recruited from childcare facilities in the district of Marburg, Middle West Germany.

Screening for child ADHD symptoms was conducted beforehand to enrich the sample with children showing high ADHD symptoms. Parents completed a screening questionnaire on their child’s ADHD symptoms (Döpfner et al. 2008) (see measures for a description). In total, 113 preschoolers who scored above the lower bound of the 95% confidence interval of the clinical cut-off score of the questionnaire (equivalent to the 70th percentile of the questionnaire) and 85 children who scored below this point were included in the study. We chose the lower bound of the 95% confidence interval of the clinical cut-off to ensure that a broad spectrum of children with ADHD-typical difficulties is examined. Exclusion criteria were: IQ < 80, motor disabilities, sensory handicaps, chronic diseases involving brain functions or the HPA axis, indication of trauma (serious physical maltreatment, life-threatening injury), any continuous pharmacological treatment, and insufficient German language skills of parents or child.

**Fig. 1:**
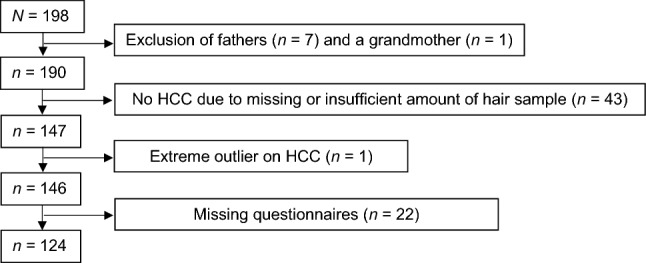
Flowchart of excluded and missing cases

The total sample consisted of 198 parent–child dyads. Since only seven fathers participated in the data collection, fathers and their children had to be excluded from the analyses to prevent potential gender bias. A dyad with a grandmother as a primary caregiver was also removed from the sample. A further 65 dyads had to be excluded due to missing data and an additional dyad was excluded because of an extreme outlier (SD = 10.31) on the HCC variable (for details, see Fig. 1). Excluded and included data did not differ with respect to child’s gender (*χ*^2^ (1) = 0.01; *p* = 0.20), educational level of mother (*χ*^2^ (5) = 5.36; *p* = 0.37), educational level of father (*χ*^2^ (3) = 4.63; *p* = 0.92), age of mother (*t*(193) = 0.95; *p* = 0.34), age of child (*t*(195) = − 0.87; *p* = 0.38), child ADHD rating scale parent rating (*t*(121.72) = 0.1; *p* = 0.99), child ADHD rating scale teacher rating (*t*(192) = − 0.48; *p* = 0.63), child ADHD symptoms clinical interview (*t*(189) = 0.27; *p* = 0.79), child ODD/CD symptoms (*t*(121.25) = 0.48; *p* = 0.64), and depressive symptoms of mother (*t*(188) = 1.84; *p* = 0.07). However, there was a significant difference with respect to maternal ADHD symptoms, as indicated by both the Conners’ Adult ADHD Rating Scale (*t*(188) = 2.33; *p* = 0.02) and the Wender–Reimherr Interview for adults (*t*(194) = 2.39; *p* = 0.02), with included mothers scoring higher (*M*_CAARS_ = 19.45; SD_CAARS_ = 10.47; *M*_WRI_ = 9.4; SD_WRI_ = 6.64) than mothers who were excluded from the analyses due to incomplete data (*M*_CAARS_ = 15.85; SD_CAARS_ = 9.42; *M*_WRI_ = 11.53; SD_WRI_ = 5.49). Thus, there was a higher probability of missing data for mothers with lower ADHD symptoms.

The final sample consisted of 124 mother–child dyads. Characteristics of the sample and comparisons between child ADHD symptom groups are presented in Table 1. The groups did not differ concerning child’s age, child’s gender, mother’s educational level, mother’s partnership status, maternal ADHD symptoms, and HCC. Significant differences were found regarding child ADHD composite score and ODD/CD symptoms, mother’s age, depression score, and reported chronic stress.

**Table 1:** Comparison of children with and without ADHD regarding child and mother demographic characteristics and study variables

	ADHD^a^	Non-ADHD	Comparison	Total sample
*n*	40	84		
Child				
Age in months *M* ± SD (range)	58.53 ± 6.03 (49 to 73)	57.52 ± 5.78 (49 to 71)	*t*(122) = − 0.89; *p* = 0.38	57.85 ± 5.86 (49 to 73)
Gender *n* (%)				
Male	20 (50%)	51 (60.7%)	χ^2^ (1) = 1.27; *p* = 0.33	71 (57.3%)
Female	20 (50%)	33 (39.3%)		53 (42.7%)
ADHD composite score *M* ± SD	2.33 ± 1.86 (− 0.88 to 8.42)	− 1.09 ± 1.41 (− 4.03 to 2.64)	*t*(122) = − 11.36; *p* < 0.01	0.1 ± 2.24 (− 4.03 to 8.42)
ODD/CD symptoms	11.24 ± 8.02 (0 to 34)	7.42 ± 5.3 (0 to 29)	*t*(55.73) = − 2.75; *p* = 0.01	8.65 ± 6.52 (0 to 34)
Mother				
Age in years *M* ± SD (range)	34.35 ± 4.88 (24–45)	36.75 ± 5.22 (24 to 47)	*t*(122) = 2.44; *p* = .02	35.96 ± 5.21 (24 to 47)
Educational level *n* (%)				
No compl. education	0	1 (1.2%)	*χ*^2^ (3) = 7.28; *p* = 0.06	1 (0.8%)
Basic education (Hauptschulabschluss)	5 (12.5%)	3 (3.6%)		8 (6.5%)
Work qualification (Realschulabschluss)	20 (50%)	28 (33.3%)		48 (38.7%)
High school	6 (15%)	18 (21.4%)		24 (19.4%)
College	9 (22.5%)	34 (40.5%)		43 (34.7%)
Living in a partnership *n* (%)	33 (82.5%)	78 (92.9%)	*χ*^2^ (1) = 3.10; *p* = 0.07	111 (89.5%)
ADHD composite score *M* ± SD (range)	0.4 ± 1.75 (− 2.75 to 4.58)	− 0.19 ± 1.82 (− 3.34 to 5.33)	*t*(122) = − 1.69; *p* = 0.09	0.01 ± 1.81 (− 3.34 to 5.33)
CES-D *M* ± SD (range)	16.27 ± 8.47 (2 to 38)	12.15 ± 8.33 (0 to 43)	*t*(122) = − 2.56; *p* = 0.01	13.48 ± 8.56 (0 to 43)
SSCS *M* ± SD (range)	23.51 ± 8.95 (5 to 40)	18.16 ± 8.65 (1 to 46)	*t*(122) = − 3.19; *p* < 0.01	19.89 ± 9.06 (1 to 46)
HCC^b^ pg/mg *M* ± SD (range)	8.20 ± 11.92 (0.91 to 62.94)	6.88 ± 7.28 (0.43 to 47.07)	*t*(122) = − 0.22; *p* = 0.83	7.31 ± 9.01 (0.43 to 62.94)
ADHD^c^ *n* (%)	3 (7.5%)	7 (8.3%)	*χ*^2^ (1) = 0.03; *p* = 0.59	10 (8.1%)

Descriptions: *N* = 124; *t* tests were conducted using bootstrap confidence intervals due to non-normal distribution of the study variables

*ADHD* attention-deficit/hyperactivity disorder, *CES-D* Centre for Epidemiologic Studies Depression Scale, *HCC* hair cortisol concentration, *ODD/CD* oppositional defiant disorder/conduct disorder, *SSCS* Screening Scale of Chronic Stress

^a^Children scoring above the 90th percentile on the ADHD symptom scale (for description see below) either in parent or teacher report

^b^*M* and SD calculated before log transformation

^c^Mothers scoring above the 93rd percentile (*T* = 65) on the Conners’ Adult ADHD Rating Scale (for description see below)

Written informed consent to participate in the study was obtained from the parents. Parents received an expense allowance of €50. Approval for the study was granted by the Ethics Committee of the Medical Faculty at the Philipps-University in Marburg.

### Procedure

The present study was a part of a larger longitudinal study with extensive data collection procedures which have been described in detail elsewhere (Pauli-Pott et al. 2017, 2019; Schloß et al. 2018). Primary caregivers were required to answer a questionnaire measuring their own and their children’s ADHD symptoms. The latter was also completed by teachers. Furthermore, primary caregivers were asked to complete additional questionnaires, including a questionnaire assessing their perceived chronic stress, depressive symptoms and ODD/CD symptoms of their children at home. Moreover, mothers were interviewed by telephone, including the interviews measuring their own and their children’s ADHD symptoms. Hair samples from the mother and the child were collected during a home visit by trained study personnel.

### Measures

#### Child ADHD symptoms

Child ADHD symptoms at the age of 4–5 years were assessed using an interview and questionnaires completed by mothers and teachers. The semi-structured clinical interview *Preschool-Parent Account of Child Symptoms* (Pre-PACS) (Daley 2010) was conducted with the mothers. Pre-PACS is a modified version of the Parental Account of Childhood Symptoms (PACS) interview (Taylor et al. 1986) for preschool children. As part of the interview, parents were asked to indicate the intensity and frequency of certain ADHD-specific behaviours of their child in the last three months. The ADHD scale of the interview shows good test–retest reliability (*r* = 0.78, after 15 weeks) and can reliably differentiate between children with and without ADHD (Sonuga-Barke et al. 2003).

In addition, the preschool version of the *ADHD rating scale* (Fremdbeurteilungsbogen für Aufmerksamkeitsdefizit-/Hyperaktivitätsstörungen im Vorschulalter, FBB-ADHS-V) (Döpfner et al. 2008) from the Diagnostic System for Mental Disorders in Childhood and Adolescence (DISYPS-II) (Döpfner et al. 2008) was completed by parents and kindergarten teachers. The FBB-ADHS-V is a 19-item questionnaire capturing the core symptoms of ADHD in preschool age, according to the ICD-10 (WHO 2016) and DSM-IV-TR (APA 2000). Items were rated on a 4-point Likert scale (from 0 = not at all to 3 = very much). Both the parent and the teacher version have demonstrated excellent internal consistency (*α*_parents_ = 0.94; *α*_teachers_ = 0.93) and good validity (distinction between children with and without ADHD) (Breuer and Döpfner 2008). The three scores intercorrelated as follows: FBB-ADHS-V parent and teacher score: *r* = 0.34 (*p* < 0.01); FBB-ADHS-V parent and Pre-PACS: *r* = 0.45 (*p* < 0.01); FBB-ADHS-V teacher and Pre-PACS: *r* = 0.16 (*p* = 0.07). A composite ADHD child symptom score, identical to that used by Pauli-Pott et al. (2017), was created by summing up the *z*-transformed scores of these three scales. The composite score showed an acceptable internal reliability (*α* = 0.60).

#### ODD/CD symptoms

Child ODD/CD symptoms at the age of 4–5 years were assessed with the German FBB-SSV questionnaire (Döpfner et al. 2008), which measures ODD/CD symptoms of children and adolescents according to the ICD-10. The scale consists of 15 items, nine of which measure ODD symptoms and six of which measure CD symptoms. Items are rated on a 4-point Likert scale (from 0 = not at all to 3 = very much). Higher scores indicate more severe ODD/CD symptoms. The scale has high-internal consistency (Cronbach’s alpha = 0.91) and validly discriminates between children with ODD/CD and controls (Gortz-Dorten et al. 2014).

#### Maternal ADHD symptoms

Maternal ADHD symptoms were assessed using the *Wender-Reimherr Interview for adults* (WRI) (Rösler et al. 2007) and the short German version of the *Conners’ Adult ADHD Rating Scale* (CAARS-K) (Christiansen et al. 2014).

The WRI is a semi-structured interview assessing adult-specific ADHD symptoms. The 28 questions of the interview which cover ADHD-related psychopathological characteristics can be assigned to the seven Wender–Utah criteria: inattention, hyperactivity, hot temperament, affective lability, emotional overactivity, disorganisation, and impulsivity. Each question is answered on a 3-point Likert scale. The interview has good reliability (*α* = 0.82) and differentiates reliably between adults with and without ADHD (Rösler et al. 2007).

The CAARS-K is a self-report questionnaire measuring adult ADHD symptoms. The total of 26 items assesses symptoms of inattention/memory problems, hyperactivity/restlessness, impulsivity/emotional lability, and problems with self-concept. Each item is rated on a 4-point Likert scale from 0 = not at all/never to 3 = very much/very frequently. The scale has shown excellent internal consistency (*α* = 0.9) and can differentiate well between adults with and without ADHD (Christiansen et al. 2014).

The two scales intercorrelated significantly (*r* = 0.64; *p* < 0.01). Analogous to the computation of the child’s ADHD score, a composite ADHD score was also created for mothers by summing up the z-transformed scores of the two scales. The composite score showed good internal reliability (*α* = 0.78).

#### Maternal depressive symptoms

The German version of the Centre for Epidemiologic Studies Depression Scale (CES-D) (Hautzinger et al. 2012) was used to assess maternal depressive symptoms. The CES-D is a 20-item screening instrument measuring depressive mood in general and clinical populations. Participants indicated on a 4-point Likert scale (from 0 = rarely or none of the time to 3 = most or all of the time) how often they had experienced symptoms associated with depression (e.g. restless sleep, poor appetite, and depressive mood) over the past week. Higher scores indicate more severe depressive symptoms, with a score above 22 indicating a high risk for clinical depression. For this version, good internal consistency (Cronbach’s *α* = 0.89) and validity (correlations with other depression questionnaires) have been established (Hautzinger et al. 2012).

#### Perceived chronic stress

The *Screening Scale of the Trier Inventory for Chronic Stress* (TICS-SSCS) (Schulz et al. 2004) was used to measure maternal perceived chronic stress. The scale provides a comprehensive global measure of experienced chronic stress and is suitable for the identification of people with high or low stress levels. The TICS-SSCS measures the frequency of experienced stress within the last three months in five different areas: chronic worrying, work overload, social overload, excessive demands and lack of social recognition. The screening scale comprises 12 items that are rated on a 5-point Likert scale from 0 = never to 4 = very often. A higher scale score corresponds to higher stress levels. In the present study the scale has shown excellent internal consistency (*α* = 0.91).

#### Hair cortisol concentration (HCC)

Mothers’ HCC was assessed using hair samples. Several thin hair strands were cut from the posterior vertex region of the head as close as possible to the scalp. HCC was determined using the first proximal three centimetres closest to the scalp, which is thought to reflect the cumulative cortisol secretion of the past three months (Wennig 2000). The HCC determination followed the laboratory protocol of Stalder et al. (Stalder et al. 2012), with minor changes. First, hair samples were washed twice in succession for 3 min in 3 ml of isopropanol. For the extraction of cortisol, 10.0 ± 0.5 mg whole, finely cut hair samples were incubated in 1.8 ml of methanol for 18 h at room temperature. After the incubation, 1.6 ml of the extract was heated at 50 °C under a constant stream of nitrogen until the methanol had evaporated and the sample was completely dry. Finally, the samples were resuspended with 150 μl gradient HPLC water (Fisher Scientific) and were vortexed for 20 s. For the determination of cortisol concentration, 50 μl extract was analysed using commercially available cortisol luminescence immunoassay (LIA; IBL International, a Tecan Group, Hamburg, Germany).

### Statistical analysis

Preliminary analyses using bivariate correlation analyses with bootstrap confidence intervals were conducted in preparation for major analyses and to examine relationships among demographic variables (child’s gender, child’s age, mother’s age, mother’s educational level), predictor variables and the two outcome variables. None of the demographic variables was related to perceived chronic stress. As log-transformed HCC correlated significantly with mother’s age, it was controlled for in the analysis of hypothesis 3.

To analyse hypothesis 1, a hierarchical multiple regression analysis was conducted with mothers’ perceived chronic stress as an outcome variable. Child and maternal ADHD symptoms were entered in step 1 and child ODD/CD and maternal depressive symptoms in step 2.

Hypothesis 2 was tested with a moderation analysis using the SPSS macro PROCESS (Hayes, 2018). Maternal chronic stress was entered as an outcome variable, child ADHD symptoms as a predictor variable and maternal ADHD symptoms as a moderator variable. Child ODD/CD and maternal depressive symptoms were also entered in the analysis as covariates. All of the predictors and covariates were mean-centered before being entered into the analysis. The analysis was conducted with a heteroscedasticity-consistent standard error estimator (HC3) to counter the violation of the assumption of homoscedasticity.

Hypothesis 3 was tested with a hierarchical regression analysis, with HCC as an outcome variable. HCC showed an extremely skewed distribution. An extreme outlier (SD = 10.31) was excluded, and log-transformation was executed to normalise the distribution. Child and maternal ADHD symptoms were entered in the first step, and child ODD/CD, maternal depressive symptoms, and mother’s age were entered in the second step.

For all three regression models, normality of residuals, linearity, multicollinearity, homoscedasticity, and the absence of influential outliers were confirmed.

## Results

Intercorrelations among study variables are depicted in Table 2. Correlation patterns were consistent with expectations. Maternal perceived chronic stress was positively correlated with child and maternal ADHD, child ODD/CD and maternal depressive symptoms. Contrary to expectations, HCC did not correlate with any of the child and maternal psychopathology measures.

**Table 2:** Intercorrelation among study variables

	1	2	3	4	5	6	7
1. Chronic stress	–						
2. HCC	0.11	–					
3. Child ADHD	0.23**	− 0.07	–				
4. ODD/CD	0.28**	− 0.10	0.40**	–			
5. Maternal ADHD	0.54**	− 0.01	0.20*	0.27**	–		
6. Depression	0.65**	0.08	0.21*	0.25**	0.45**	–	
7. Mother’s age	− 0.12	0.27**	− 0.19*	− 0.01	− 0.06	− 0.15	–

Significance test for Pearson’s correlation coefficients were estimated using bootstrap confidence intervals

*p < 0.05; **p < 0.01

The first regression model, examining the impact of child and maternal ADHD symptoms on maternal perceived chronic stress while controlling for child ODD/CD and maternal depressive symptoms, was significant (*F*(4, 119) = 30.24; *p* < 0.01) and accounted for 50% of the variance in maternal perceived chronic stress. Regression coefficients are depicted in Table 3. The introduction of child and maternal ADHD symptoms in step 1 of the equation led to a significant prediction of mothers’ chronic stress (*R*^2^ = 0.31; *F*(2, 121) = 27.15; *p* < 0.01), but only the effect of maternal ADHD symptoms was uniquely significant (*β* = 0.52, *p* < 0.01). The introduction of child ODD/CD and depressive symptoms significantly improved the prediction of maternal perceived chronic stress (Δ*R*^2^ = 0.19; Δ*F*(2, 119) = 23.31; *p* < 0.01). Only the contribution of maternal depressive symptoms was uniquely significant (*β* = 0.49; *p* < 0.01). For partial regression plots see supplementary information.

The moderation term of the analysis exploring the moderating role of maternal ADHD symptoms in the association between reported child ADHD symptoms and maternal perceived chronic stress was not significant (*b* = − 0.01; SE *b* = 0.17; *t*(5, 118) = − 0.05; *p* = 0.96), indicating no significant moderation effect.

The third regression model, predicting mother’s HCC by child and maternal ADHD symptoms while controlling for child ODD/CD and maternal depressive symptoms as well as mother’s age, reached significance (*F*(5, 118) = 2.73; *p* = 0.02) and accounted for 10% of the variance in mother’s HCC (Table 4). Neither child nor maternal psychopathology accounted for any variance in HCC. Only mother’s age contributed significantly to the explanation of the variance in mother’s HCC (*β* = 0.29; *p* < 0.01). The positive β*-*value suggests that mothers’ HCC increased with age.

## Discussion

The present study is the first to test whether children’s ADHD symptoms affect their mothers’ perceived chronic stress, while simultaneously considering maternal ADHD and child ODD/CD as well as maternal depressive symptoms. A further unique contribution of this study lay in the inclusion of HCC to assess the cumulative secretion of cortisol as a long-term measure of the stress-sensitive HPA axis activity.

Contrary to our expectations, child ADHD symptoms did not account for significant variance in maternal perceived chronic stress when maternal ADHD, child ODD/CD, and maternal depressive symptoms were also considered. Most importantly, our findings suggest that maternal factors are stronger predictors of maternal perceived chronic stress than child-level factors. Maternal ADHD symptomatology did not emerge as a moderator of the association between child ADHD symptoms and maternal perceived chronic stress, and nor did child and maternal psychopathology factors account for variance in maternal HCC.

The study makes several contributions to the literature regarding negative effects of child ADHD symptoms on mothers’ psychosocial functioning, by demonstrating that perceived chronic stress of mothers is affected rather by their own psychopathology than by that of their children. This finding is partly in contrast to studies reporting considerable negative effects of child ADHD on mothers’ parenting stress (DuPaul et al. 2001; Johnston and Mash 2001; Theule et al. 2013). This discrepancy can partly be explained by the fact that most studies on parenting stress focussed mainly on the effects of child ADHD symptomatology, and failed to consider the influence of other relevant factors such as ODD/CD or maternal psychopathology (Johnston and Mash 2001; Theule et al. 2013). The majority of the studies which considered either child ODD/CD or maternal psychopathology also demonstrated that not ADHD itself, but rather these other factors, are primarily responsible for mothers’ elevated parenting stress (Schworer et al. 2020; Theule et al. 2011). In contrast to these studies, however, we found that none of the child-level factors contributed to mothers’ perceived chronic stress beyond maternal ADHD and depressive symptoms. This may be due to the inherent differences in the constructs of parenting stress and chronic stress: parenting stress arises from distress experienced by parenting-related demands (Abidin 1995), while chronic stress, as defined in this study, captures excessive general stress experienced by a person in different life domains, such as work or general social contexts (Schulz et al. 2004). Thus, it seems rather conclusive that this general stress is affected more by mothers’ own characteristics than by the characteristics of their children.

Nevertheless, qualitative studies on the experience of mothers of children with ADHD suggest that having a child with ADHD has a considerable influence on their resources (Sikirica et al. 2015), making successful coping difficult for this population. Thus, it is still somewhat surprising that child ADHD symptoms did not account for significant variance beyond maternal characteristics in the present study. A potential explanation may lie in the relatively young age of the children included in our sample: it is conceivable that critical transitions such as school entry and the age-related trajectory of ADHD may influence maternal perceived stress over time. For example, while school entry is stressful per se for both child and parent, new demands and expectations (e.g., sitting still, doing homework) are particularly challenging for children with ADHD. Within the demanding school context, ADHD symptoms become more palpable, which, in turn, may contribute to maternal perceived stress over time (Banaschewski et al. 2017; Feldman and Reiff 2014). Longitudinal studies are needed to gain a better understanding of maternal stress over time in mothers of children with ADHD.

Mothers’ ADHD symptoms, on the other hand, exerted a relatively large effect on their perceived chronic stress. This finding is in line with previous studies linking adult ADHD symptoms to higher general stress (Combs et al. 2015; Hirvikoski et al. 2009). Given the subclinical nature of the sample (only about 8% of the mothers reached the diagnostic threshold), the relatively large effect of maternal ADHD symptoms is even more compelling.

Depressive symptomatology of mothers also proved to be a strong predictor of maternal perceived chronic stress in this study. This is in line with previous studies identifying maternal depression as the strongest predictor of mothers’ parenting stress (Biondic et al. 2019; Schworer et al. 2020). A strong association between chronic stress and depression has been well-established in previous studies (Hammen 2005).

Nevertheless, this finding should be interpreted with caution: due to the correlational design of the study, it is not possible to derive implications about causation. Although individuals with depressive symptoms might experience more stress in their lives than those without or with mild depressive symptoms (Hammen 2005), the majority of psychological models of depression consider stress as a triggering risk factor for depression, and not vice versa (Phillips et al. 2015; Scher et al. 2005). The mutual influence between the two variables should also be taken into consideration. Longitudinal studies are needed to understand the nature of this relationship.

The present findings did not support either the similarity-fit or the similarity-misfit hypothesis. The reason for the lack of significant moderation effect may lie in the substantial amount of variance explained by maternal ADHD and depressive symptoms, possibly leaving only little variance to be explained by the interaction effect. The similarity-fit or the similarity-misfit approach, which originally addressed the interaction effect of child and parental ADHD on parenting behaviour, might not be easily transferable to parental stress.

The final aim of the study was to analyse the effect of child and maternal ADHD and child ODD/CD as well as maternal depressive symptoms on HCC. None of these variables were associated with maternal HCC in the present study. In contrast to previous findings of hypocortisolism in caregivers of children with a psychological disorder; schizophrenia: (Gonzalez-Bono et al. 2011); autism: (Radin et al. 2019; Seltzer et al. 2010); eating disorders: (Ruiz-Robledillo et al. 2016), providing care for a child with ADHD was not associated with altered HPA axis activity in this study. There are several possible explanations for this result.

First, the lack of association may be due to the young age of the children included in this study. With the exception of Radin et al. (2019), who examined children between the ages of 2 and 16 years, previous studies exclusively included caregivers of adolescent and adult offspring (Gonzalez-Bono et al. 2011; Seltzer et al. 2010; Ruiz-Robledillo et al. 2016). According to the model of allostatic load, physiological changes induced through chronic stress mostly occur only after a long-standing, intensive stress load (McEwen 1998). Thus, mothers of preschool children might not yet have been overwhelmed (long) enough for physiological changes to occur. Furthermore, the parents examined in previous studies were older than the mothers in the current study ((Gonzalez-Bono et al. 2011), [*M*_age_ = 63.61]; (Seltzer et al. 2010), [*M*_age_ = 53.9]; (Ruiz-Robledillo et al. 2016), [*M*_age_ = 50.86]). Older individuals typically have poorer health (Lovell and Wetherell 2011), which may make them more vulnerable to stress-related physiological changes.

Another explanation for this divergent result may lie in the means of cortisol measurement. Most of the studies conducted with informal caregivers used salivary cortisol (Gonzalez-Bono et al. 2011; Seltzer et al. 2010; Ruiz-Robledillo et al. 2016), except (Radin et al. 2019) using HCC). Thus, the significant findings of these previous studies may be attributable to measurement errors inherent in the use of salivary cortisol to assess long-term HPA axis activity (Stalder et al. 2017).

Moreover, maternal ADHD symptoms were not associated with HCC either. Previous studies likewise failed to find any associations between adult ADHD symptoms and HPA axis hypoactivity using salivary cortisol measures (Bonvicini et al. 2016; Hirvikoski et al. 2009; Ramos-Quiroga et al. 2016). Nevertheless, our finding partially contradicts previous studies reporting associations between child ADHD symptoms and HPA hypoactivity (Isaksson et al. 2015; Scassellati et al. 2012). It is conceivable that gender plays a role in this association: while the majority of the studies conducted with children solely included boys, the gender ratio in studies with adults was evenly balanced. Thus, ADHD may only be associated with HPA hypoactivity in men and not in women. This assumption is further supported by a study which found a significant association between ADHD symptoms and HCC in boys but not in girls (Pauli-Pott et al. 2019).

Given the relatively small number of mothers who scored above the diagnostic threshold for ADHD in the present study, the lack of significant association between maternal ADHD symptoms and HCC might also have been due to the subclinical nature of the sample (Tables 3, 4).

**Table 3:** Hierarchical multiple regression predicting chronic stress of mothers from child and maternal ADHD symptoms while controlling for child ODD/CD and maternal depressive symptoms

Steps and predictors	Estimate	SE	95% Cl		*t*	*p*
			LL	UL		
Step 1						
Constant	19.88	0.68	18.53	21.23	29.16	< 0.01
Child ADHD	0.54	0.31	− 0.08	1.15	1.71	0.09
Maternal ADHD	2.58	0.39	1.81	3.34	6.69	< 0.01
Step 2						
Constant	12.29	1.41	9.49	15.08	8.71	< 0.01
Child ADHD	0.21	0.29	− 0.36	0.78	0.73	0.46
Maternal ADHD	1.49	0.37	0.76	2.22	4.06	< 0.01
ODD/CD	0.7	0.1	− 0.13	0.27	0.71	0.48
Depression	0.52	0.08	0.36	0.67	6.67	< 0.01

*Cl* confidence interval, *LL* lower limit, *UL* upper limit

*R*^2^ = 0.31; *F*(2, 121) = 27.15; *p* < 0.01; for step 1; Δ*R*^2^ = 0.19; Δ*F*(2, 119) = 23.31; *p* < 0.01 for Step 2; *n* = 124

**Table 4:** Hierarchical multiple regression predicting HCC of mothers from child and maternal ADHD symptoms while controlling for child ODD/CD and maternal depressive symptoms and mother’s age

Steps and predictors	Estimate	SE	95% Cl		*t*	*p*
			LL	UL		
Step 1						
Constant	4.84	0.16	4.16	5.64	20.48	< 0.01
Child ADHD	1.03^a^	0.02	1.1^a^	1.04	− 0.79	0.43
Maternal ADHD	1	0.02	1.09^a^	1.09	0.03	0.97
Step 2						
Constant	1.23^a^	0.3	3.76^a^	2.49	− 0.37	0.72
Child ADHD	1	0.02	1.07^a^	1.08	0.07	0.95
Maternal ADHD	1.02^a^	0.02	1.11^a^	1.08	− 0.35	0.72
ODD/CD	1.02^a^	0.01	1.04^a^	1.01	− 1.37	0.17
Depression	1.02	0.01	1.01^a^	1.04	1.72	0.09
Mother’s age	1.05	0.01	1.02	1.08	3.23	< 0.01

Descriptions: *R*^2^ = 0.01; *F*(2, 121) = 0.32; *p* = .72; for step 1; Δ*R*^2^ = 0.09; Δ*F*(3, 118) = 4.32; *p* = 0.01 for Step 2; *n* = 124

*Cl* confidence interval, *LL* lower limit, *UL* upper limit

^a^Values were negative before back-transformation

According to our findings, mothers’ age was the only significant predictor of HCC, with HCC increasing with age. This is in line with previous studies using plasma cortisol (VanCauter et al. 1996; Deuschle et al. 1997), suggesting an age-related increase in cortisol secretion. Results with HCC have shown a more inconsistent picture (Wosu et al. 2013), with some studies finding a positive but weak relationship between HCC and age (Dettenborn et al. 2012; Stalder et al. 2017) and others failing to find a significant association (Dettenborn et al. 2010; Manenschijn et al. 2011). This inconsistency may be explained by study findings indicating a more pronounced increase in cortisol secretion with age in women than in men (Otte et al. 2005; VanCauter et al. 1996). In contrast to the two studies mentioned above (Dettenborn et al. 2010; Manenschijn et al. 2011), the present work examined only women, which might have made the effect of age on cortisol secretion more apparent. Nevertheless, the relationship between HCC and mother’s age is still noteworthy considering the relatively narrow age range (24–47 years) of the women in our study.

Furthermore, the age-related increase in cortisol secretion in previous studies was primarily observed in somewhat older participants (> 50 years) (Dettenborn et al. 2012; Larsson et al. 2009; VanCauter et al. 1996). The result of the present study thus indicates that increased cortisol secretion is already observable in younger women. However, there is also a possibility that this association was driven not by age-related physiological changes but rather by mothers’ increased stress levels caused by the increasing burden with age (more children, greater workload, elderly parents in need of care) or by the chronicity of their ADHD symptoms.

The present study has several limitations. The percentage of mothers with clinically relevant ADHD symptoms was quite low. Nevertheless, the dimensional approach of the current study showed an association between maternal ADHD symptoms and perceived chronic stress. Furthermore, the assessment of maternal ADHD symptoms was based exclusively on self-report. This calls the validity of our results into question, as adults with ADHD tend to underestimate their symptoms (Guelzow et al. 2017; Sibley et al. 2012). For a reliable diagnosis of ADHD in adulthood, a retrospective assessment of ADHD symptoms in childhood is also required (Kooij et al. 2019).

Unfortunately, for statistical reasons, fathers had to be excluded from the current analyses because the majority of participating primary caregivers in this study were mothers; hence, it was not possible to generalise the results to fathers or to draw comparisons between mothers and fathers. Further work examining both mothers and fathers is needed to ascertain whether child ADHD symptoms impact their chronic stress differently. Further research should also investigate whether and how ADHD symptoms of the partner have an impact on the co-parent besides the ADHD symptoms of a child. Furthermore, given that social support provided by a partner or another family member as well as the number of children in the family might also (negatively or positively) affect maternal stress. Future studies are needed to examine the role of these contextual variables.

A general limitation of this study lies in its correlational nature, making it impossible to infer causal associations. Longitudinal studies could be able to clarify this matter.

## Conclusions

The present study goes some way towards improving our understanding of the factors influencing chronic stress in mothers of children with elevated ADHD symptoms. Furthermore, the study is the first to investigate the similarity-fit/misfit process in the context of chronic stress.

Taken together our findings add to the literature by revealing that maternal own ADHD and depressive symptoms significantly contribute to self-reported stress, while child’s ADHD and ODD/CD symptomatology may play only a minor role in these vulnerable mothers. Furthermore, the relationship between child ADHD symptoms and maternal chronic stress did not change with increasing maternal ADHD symptoms. These findings from self-report measures were not supported by a biological stress measure.

Thus, treating children with ADHD should include heightened awareness for subthreshold depression and ADHD related problems of mothers, by either referring them to an adult specialist or revising treatment manuals for children with ADHD including work with parents going beyond the honing parenting skills (e.g. stress management).

## Supplementary Information

Below is the link to the electronic supplementary material.

Supplementary file1 (DOCX 238 kb)

## Footnotes

1 Informal or familial caregivers provide unpaid ongoing assistance to a family member with a chronic condition. They should be distinguished from formal or professional caregivers who get paid for their professional services (Roth et al. 2015).
