# Supplementary material for: The impact of preschool child and maternal attention-deficit/hyperactivity disorder (ADHD) symptoms on mothers’ perceived chronic stress and hair cortisol
Source: J Neural Transm (Vienna). 2021 Jul 6;128(9):1311–24. doi: 10.1007/s00702-021-02377-1 (PMC8423631; doi:10.1007/s00702-021-02377-1)

**Supplementary Information**

Article title: The impact of preschool child and maternal attention-deficit/hyperactivity disorder (ADHD) symptoms on mothers’ perceived chronic stress and hair cortisol

Journal name: Journal of Neural Transmission

Author names: Anna Szép, Nadine Skoluda, Susan Schloß, Katja Becker, Ursula Pauli-Pott, Urs M. Nater

Affiliation and e-mail address of the corresponding author: Urs M. Nater; Department of Clinical & Health Psychology, Faculty of Psychology, University of Vienna, Vienna, urs.nater@univie.ac.at

**Fig. 1**

*Partial Regression Plot of Self-Reported Chronic Stress and Child ADHD Symptoms*


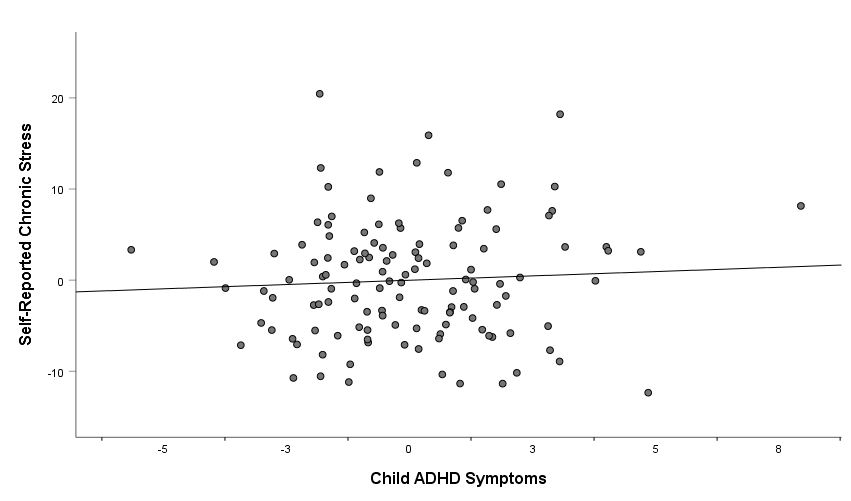


**Fig. 2**

*Partial Regression Plot of Self-Reported Chronic Stress and Maternal ADHD Symptoms*


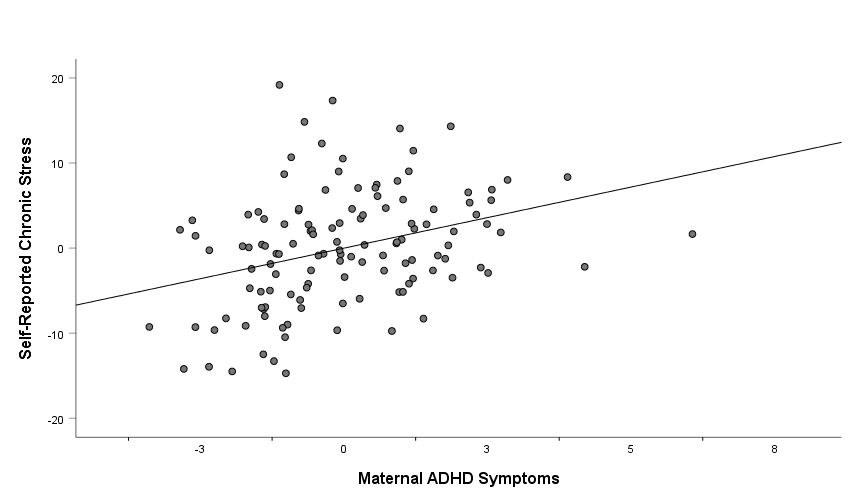


**Figure 3**

Partial Regression Plot of Self-Reported Chronic Stress and Child ODD/CD Symptoms


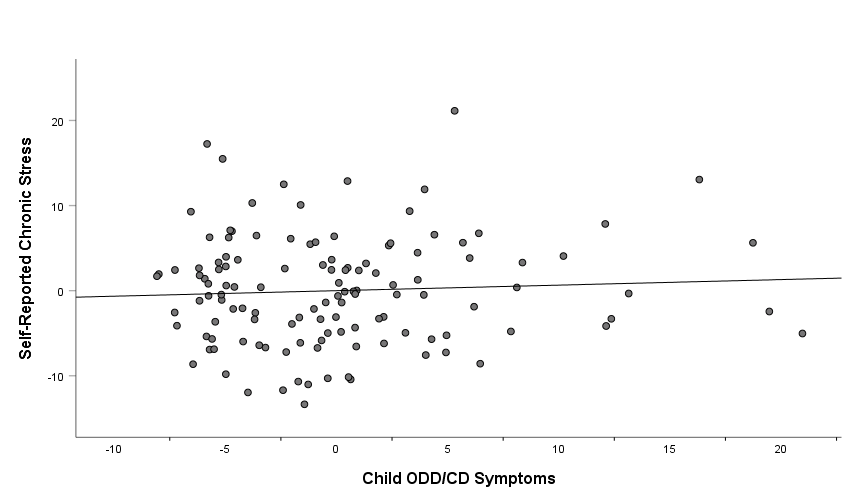


**Figure 4**

Partial Regression Plot of Self-Reported Chronic Stress and Maternal Depressive Symptoms


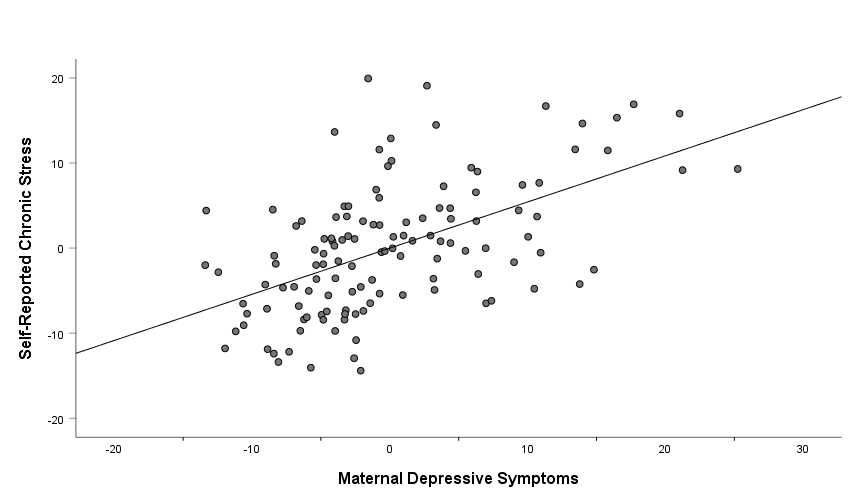


**Figure 5**

Partial Regression Plot of HCC and Child ADHD Symptoms


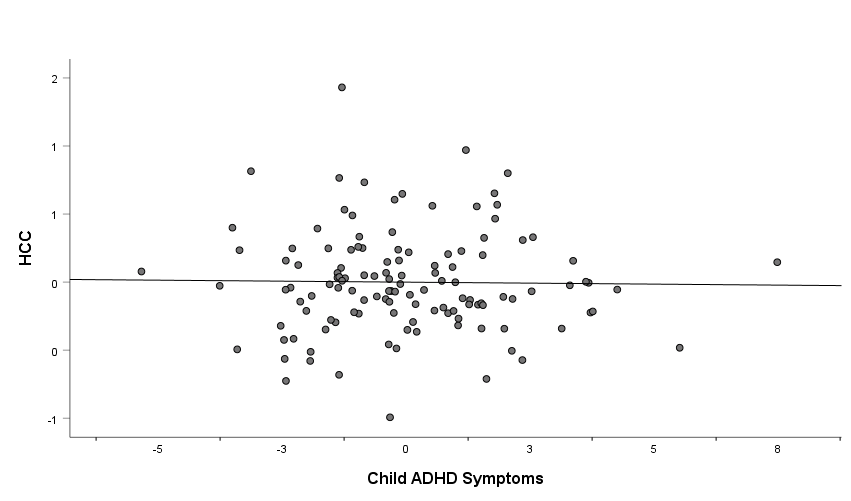


**Figure 6**

Partial Regression Plot of HCC and Maternal ADHD Symptoms


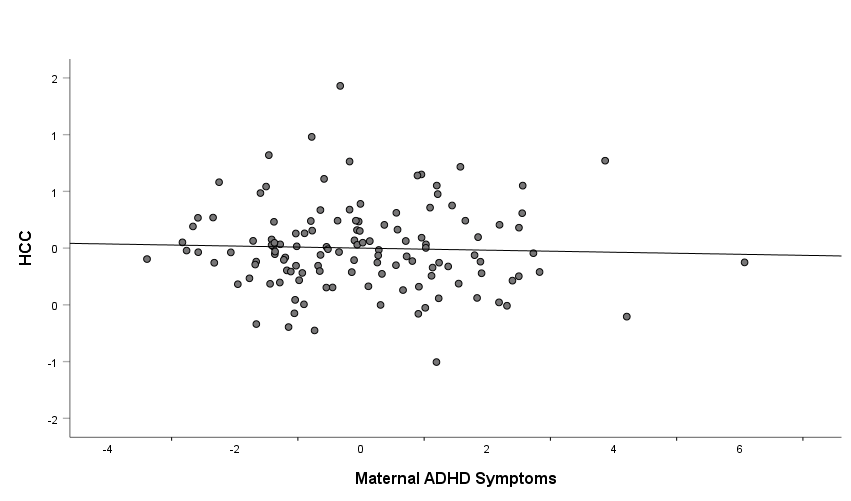


**Figure 7**

Partial Regression Plot of HCC and Child ODD/CD Symptoms


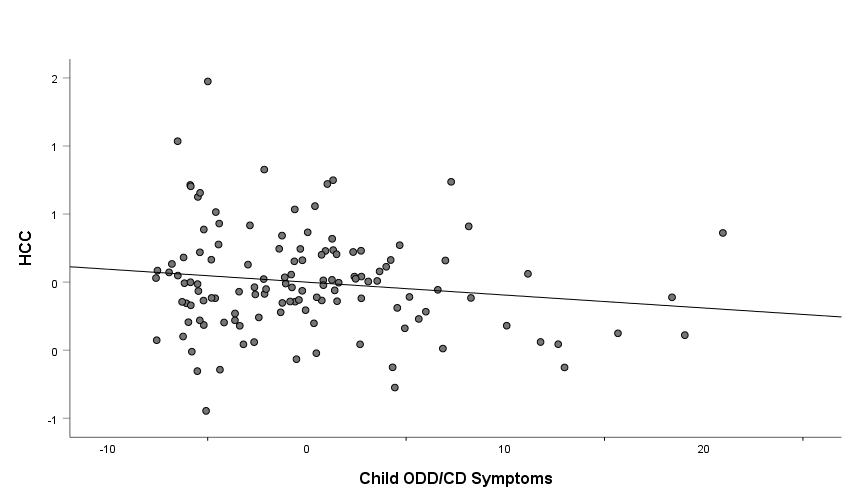


**Figure 8**

Partial Regression Plot of HCC and Maternal Depressive Symptoms


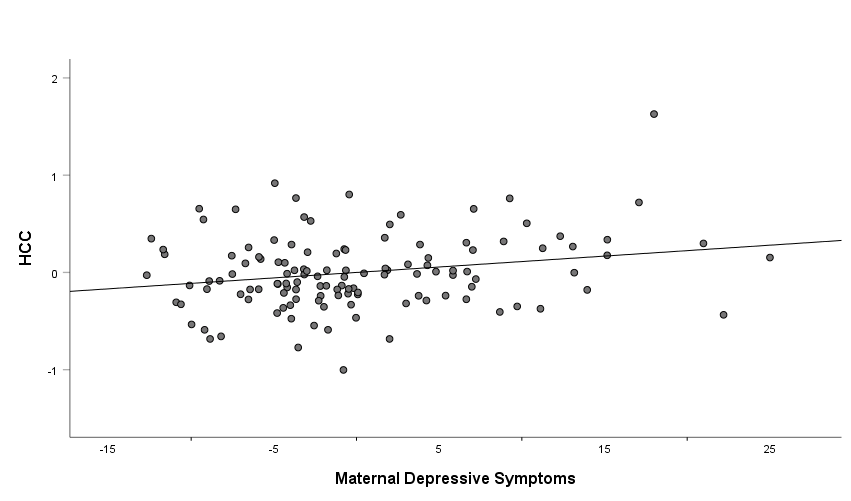


**Figure 9**

Partial Regression Plot of HCC and Mother´s Age


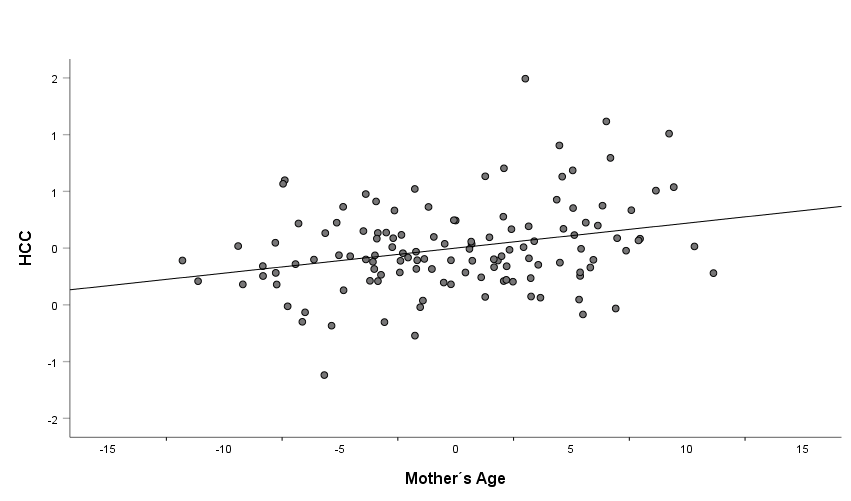

Supplement: Supplementary file 1 — Supplementary file1 (DOCX 238 kb) [file 702_2021_2377_MOESM1_ESM.docx]
